# Supplementary material for: Spatiotemporal analysis of the first wave of COVID-19 hospitalisations in Birmingham, UK
Source: BMJ Open. 2021 Oct 3;11(10):e050574. doi: 10.1136/bmjopen-2021-050574 (PMC8490999; doi:10.1136/bmjopen-2021-050574)
Supplement: Supplementary data [file bmjopen-2021-050574supp001.pdf]

SUPPLEMENTARY MATERIAL

The area of interest is $A \subset \mathbb{R}^2$ and the time period of interest is $T \subset \mathbb{R}_{\geq 0}$. The data are assumed to be realisations of a Log Gaussian Cox process (LGCP), i.e. an inhomogeneous Poisson process with stochastic intensity function $\{\lambda(s,t): s \in A, t \in T\}$.[1] The number of cases occurring in any locally finite random set $S \subseteq A$ is Poisson distributed conditional on $\lambda(s,t)$:

$$Y(S,t) = Poisson\left(\int_S \lambda(s,t)ds\right)$$

The intensity of the Poisson process is decomposed as the log-linear model:

$$\lambda(s,t) = e(s)\exp\big(\beta_0 + \beta_1 x(s,t) + Z(s,t)\big). \qquad (1)$$

In equation (1):

1. $e(s)$ is an offset, the population density (number per hectare) at location *s*; we assumed that changes within the time-window under consideration were negligible.
2. $x(s,t)$ is a set of spatially or temporally varying covariates; no covariates that varied both spatially and temporally on the temporal scale of our analyses were identified.
3. $\{Z(s,t): s \in A, t \in T\}$ is a zero-mean spatio-temporal Gaussian process with minimally parameterised covariance function:

$$Cov\big(Z(s,t), Z(s',t')\big) = \sigma^2 \rho_s\big(||s-s'||;\phi\big)\rho_t\big(||t-t'||;\theta\big)$$

where $||.||$ is the Euclidean norm.

We used a double- exponential correlation function:

$$\rho_s\big(||s-s'||;\phi\big) = \exp\left(-\frac{||s-s'||}{\phi}\right); \;\; \rho_t\big(||t-t'||;\theta\big) = \exp\left(-\frac{||t-t'||}{\theta}\right)$$

where ϕ and θ are the spatial and temporal correlation range parameters.

There are a variety of approaches to fitting LGCPs. Almost all of the computational methods discretize the area of interest using a fine regular lattice onto which case counts are aggregated.[1, 2] To ensure the reliability of the discretization, the lattice is

generally assumed to be fine enough so that the latent Gaussian field can be assumed to be approximately constant within each cell. A finer lattice would be computationally inefficient, a less fine lattice may smooth over important variation and preclude predictions at finer spatial scales. Based on likely social interactions, disease transmission, and the scales on which variation could be meaningfully interpreted, we selected a cell size of 0.005 on a longitude/latitude scale, which is approximately 500m^2. This results in a grid with 1,411 cells inside the boundary of our area of interest.

There have been few head-to-head comparisons of computational methods for LGCPs, and those that do exist consider spatial-models only.[3, 4] The principle approaches are Bayesian: a Markov Chain Monte Carlo (MCMC) approach, and a Gauss-Markov Random Field approximation of the model above estimated using MCMC or Integrated Nested Laplacian Approximation. We conducted a scoping comparison of these three approaches using simulated spatio-temporal case data and available software, and in conjunction with previous comparisons, selected a MCMC approach based on the full model as the best performing in terms of stability, computational time, and predictive accuracy. The software package lgcp for R implements the MCMC sampler for this model.[5, 6]

For the first day's analysis we used weakly informative N(0,5^2) priors on parameters in the linear predictor and for the log parameters in the covariance function. Weakly informative priors were preferred to "uninformative" priors as they provide a degree of computational stability and regularisation, while providing little information on parameter location within a plausible range for each parameter. For each subsequent day's analysis, the priors were set to the posteriors from the previous day.

REFERENCES

1. Diggle PJ, Moraga P, Rowlingson B, Taylor BM. Spatial and Spatio-Temporal Log-Gaussian Cox Processes: Extending the Geostatistical Paradigm. Stat Sci. 2013;28:542–63. doi:10.1214/13-STS441.

2. Møller J, Syversveen AR, Waagepetersen RP. Log Gaussian Cox processes. Scand J Stat. 1998;25:451–82.

3. Teng M, Nathoo F, Johnson TD. Bayesian computation for Log-Gaussian Cox processes: a comparative analysis of methods. J Stat Comput Simul. 2017;87:2227–52. doi:10.1080/00949655.2017.1326117.

4. Taylor BM, Diggle PJ. INLA or MCMC? A tutorial and comparative evaluation for spatial prediction in log-Gaussian Cox processes. J Stat Comput Simul. 2014;84:2266–84. doi:10.1080/00949655.2013.788653.

5. Taylor BM, Davies TM, Rowlingson BS, Diggle PJ. Lgcp: Inference with spatial and spatio-temporal log-gaussian cox processes in R. J Stat Softw. 2013;52:1–40.

6. Taylor BM, Davies TM, Rowlingson BS, Diggle PJ. Bayesian Inference and Data Augmentation Schemes for Spatial, Spatiotemporal and Multivariate Log-Gaussian Cox Processes in R. J Stat Softw. 2015;63. doi:10.18637/jss.v063.i07.
