# Supplementary material for: Spatiotemporal analysis of the first wave of COVID-19 hospitalisations in Birmingham, UK
Source: BMJ Open. 2021 Oct 3;11(10):e050574. doi: 10.1136/bmjopen-2021-050574 (PMC8490999; doi:10.1136/bmjopen-2021-050574)
Supplement: Author's manuscript [file bmjopen-2021-050574.draft_revisions.pdf]

BMJ Open is committed to open peer review. As part of this commitment we make the peer review history of every article we publish publicly available.

When an article is published we post the peer reviewers' comments and the authors' responses online. We also post the versions of the paper that were used during peer review. These are the versions that the peer review comments apply to.

The versions of the paper that follow are the versions that were submitted during the peer review process. They are not the versions of record or the final published versions. They should not be cited or distributed as the published version of this manuscript.

BMJ Open is an open access journal and the full, final, typeset and author-corrected version of record of the manuscript is available on our site with no access controls, subscription charges or pay-per-view fees (http://bmjopen.bmj.com).

If you have any questions on BMJ Open's open peer review process please email info.bmjopen@bmj.com

BMJ Open

Spatio-temporal analysis of the first wave of Covid-19 hospitalisations in Birmingham, UK

Journal:	*BMJ Open*
Manuscript ID	bmjopen-2021-050574
Article Type:	Original research
Date Submitted by the Author:	23-Feb-2021
Complete List of Authors:	Watson, Samuel; University of Birmingham, Institute of Applied Health Research<br>Diggle, Peter; Lancaster University, Faculty of Health and Medicine; University of Liverpool, Epidemiology and Population Health<br>Chipeta, Michael; University of Oxford<br>Lilford, RJ; University of Birmingham,
Keywords:	COVID-19, Infection control < INFECTIOUS DISEASES, STATISTICS & RESEARCH METHODS

SCHOLARONE™
Manuscripts

I, the Submitting Author has the right to grant and does grant on behalf of all authors of the Work (as defined in the below author licence), an exclusive licence and/or a non-exclusive licence for contributions from authors who are: i) UK Crown employees; ii) where BMJ has agreed a CC-BY licence shall apply, and/or iii) in accordance with the terms applicable for US Federal Government officers or employees acting as part of their official duties; on a worldwide, perpetual, irrevocable, royalty-free basis to BMJ Publishing Group Ltd ("BMJ") its licensees and where the relevant Journal is co-owned by BMJ to the co-owners of the Journal, to publish the Work in this journal and any other BMJ products and to exploit all rights, as set out in our licence.

The Submitting Author accepts and understands that any supply made under these terms is made by BMJ to the Submitting Author unless you are acting as an employee on behalf of your employer or a postgraduate student of an affiliated institution which is paying any applicable article publishing charge ("APC") for Open Access articles. Where the Submitting Author wishes to make the Work available on an Open Access basis (and intends to pay the relevant APC), the terms of reuse of such Open Access shall be governed by a Creative Commons licence – details of these licences and which Creative Commons licence will apply to this Work are set out in our licence referred to above.

Other than as permitted in any relevant BMJ Author's Self Archiving Policies, I confirm this Work has not been accepted for publication elsewhere, is not being considered for publication elsewhere and does not duplicate material already published. I confirm all authors consent to publication of this Work and authorise the granting of this licence.

For peer review only

Spatio-temporal analysis of the first wave of Covid-19 hospitalisations in Birmingham, UK

Samuel I Watson*, Senior Lecturer, Institute of Applied Health Research, University of Birmingham

Peter J Diggle, Distinguished Professor of Statistics, Lancaster University

Michael G Chipeta, Research Fellow, University of Oxford

Richard J Lilford, Professor of Public Health, Institute of Applied Health Research, University of Birmingham

*Corresponding author

For peer review only

Keywords: Disease surveillance, Covid-19, infectious disease, spatio-temporal modelling

ABSTRACT (243/300 words)

Objectives To evaluate the spatio-temporal distribution of the incidence of Covid-19 hospitalisations in Birmingham, UK during the first wave of the pandemic to support the design of public health disease control policies.

Design A geospatial statistical model was estimated as part of a real-time disease surveillance system to predict local daily incidence of Covid-19.

Participants All hospitalisations for Covid-19 to University Hospitals Birmingham NHS Foundation Trust between February 1st 2020 and September 30th 2020.

Outcome measures Predictions of the incidence and cumulative incidence of Covid-19 hospitalisations in local areas, its weekly change, and identification of predictive covariates.

Results Peak hospitalizations occurred in the first and second weeks of April 2020 with significant variation in incidence and incidence rate ratios across the city. Population age, ethnicity, and socioeconomic deprivation were strong predictors of local incidence. Hospitalisations demonstrated strong day of the week effects with fewer hospitalizations (10-20% less) at the weekend. There was low temporal correlation in unexplained variance. By day 50 at the end of the first lockdown period the top 2.5% of small areas had experienced five times as many cases per 10,000 population as the bottom 2.5%.

Conclusions Local demographic factors were strong predictors of relative levels of incidence and can be used to target local areas for disease control measures. The real-time disease surveillance system provides a useful complement to other surveillance approaches by producing real-time, quantitative and probabilistic summaries of key outcomes at fine spatial resolution to informdisease control programmes.

For peer review only

STRENGTHS AND LIMITATIONS OF THIS STUDY

- Geospatial statistical methods can provide daily predictions of disease epidemiology at small spatial scales as a complement to large-scale designed studies that report infrequently.
- Many countries and local authorities possess the data necessary to run real-time surveillance software that can readily be deployed on a desktop computer, but careful consideration of key model parameters is required.
- Understanding the key predictors and extent of spatial and temporal correlation of disease incidence and transmission can help target local case area targeted intervention (CATI) programmes.
- We report the output of a proof-of-principle surveillance system using geospatial statistical methods to predict local, daily Covid-19 risk using data on hospitalisations for Covid-19 in Birmingham, UK during the first wave of the pandemic in 2020.

For peer review only

ADDITIONAL INFORMATION

Competing interests

None declared.

Funding

UKRI/DHSC COV0036. The funders had no role in study design, data collection and analysis, decision to publish, or preparation of the manuscript.

Author contributions

SW, RJL, PJD, and MGC conceived of the research idea. SW led the development of the software, statistical analyses, and manuscript writing, with support from RJL, PJD, and MGC, who edited and approved the final version of the manuscript.

Ethical approval

Ethical approval for use of the hospital admissions data presented in this article was granted by HRA and HCRW (IRAS 288478).

Transparency declaration

The lead author* affirms that this manuscript is an honest, accurate, and transparent account of the study being reported; that no important aspects of the study have been omitted; and that any discrepancies from the study as planned (and, if relevant, registered) have been explained.

Data sharing statement

Data are not available for sharing from this project. All statistical code and documentation is publicly available at the links provided in the article.

Patient and Public Involvement

Patients were not involved in the design of this study.

Dissemination Declaration

Software and documentation of the detailed methods has been made publicly and freely available.

The Corresponding Author has the right to grant on behalf of all authors and does grant on behalf of all authors, an exclusive licence (or non exclusive for government employees) on a worldwide basis to the BMJ Publishing Group Ltd to permit this article (if accepted) to be published in BMJ editions and any other BMJPGL products and sublicences such use and exploit all subsidiary rights, as set out in our licence.

For peer review only

INTRODUCTION

A range of preventative public health measures have been deployed to limit the transmission of Covid-19 during the pandemic in 2020 and beyond. Perhaps most prominently "lockdowns" of whole regions or nations, involving the closure of businesses and public spaces and requiring people to stay home, have been implemented worldwide. Lockdowns have been effective, but are blunt instruments, with potentially large collateral social and economic effects.[1] More localised approaches that target high-risk areas and emerging disease clusters could potentially ameliorate some of these social and economic effects while maintaining low incidence rates, as has been demonstrated for other infectious diseases. For example, Ratnayake *et al* review the use of case-area targeted intervention (CATI) for cholera, which entails targeting a range of measures at small areas (50-100 meters), including chemoprophylaxis, water treatment, and vaccination, in response to the early detection of an outbreak.[2] However, the effective use of CATI requires both reliable early cluster detection and a good understanding of the spatial and temporal distribution of cases and clusters in order to delineate "case-areas".

There are a growing number of explicit or direct geospatial statistical analyses of Covid-19 transmission and spread.[3] Many of these examples use case data aggregated to small area levels (such as local government authorities or provinces) (e.g.[4,5], see [3] for a review) and so cannot provide insight into the variability of Covid-19 incidence at more local levels, across urban areas. Therefore, there is almost no published evidence or existing reporting systems that can guide CATI-type approaches for Covid-19. There are many "indirect analyses" that compare local or larger-scale risk factors with Covid-19 epidemiology, which could be used to identify high-risk areas for disease control intervention.[3,6,7] For example, population age has consistently emerged as a key risk factor in a number of studies.[8] In high income countries, more socioeconomically deprived areas and areas with a high minority ethnic population proportion have also been identified as high risk.[9] But it is not known just how well these factors explain small-scale spatio-temporal variance in incidence rates.

Geospatial statistical analysis is increasingly in use as a tool for disease surveillance, mapping, and epidemiology.[10] Incident cases and disease testing occur at discrete locations in space and time. Where data on these outcomes are spatially-referenced and time-stamped, they can be modelled to provide predictions of prevalence or incidence over an area of interest. Models can incorporate relevant covariate data and allow for spatial and temporal correlation so that predictions are "smoothed" over time and space. One specific application of these methods is for real-time surveillance.[11] Daily incident case data can be used to predict incidence rates across an area of interest, such as a city, in "real-time" to identify areas

For peer review only

with a high probability of high or rising risk.[12] We developed a proof-of-principle real-time surveillance system using individual-level data on Covid-19 hospitalisations in Birmingham, UK, although the software and statistical approach could be used for any disease.[13] The system is designed to rerun the analyses with each new day's case data, thus its output represents an evolving spatio-temporal analysis of Covid-19 hospitalisations over the course of the pandemic. This article presents results from this analysis for the first wave of the Covid-19 pandemic.

METHODS

The analyses presented in this article were conducted as part of a project to develop software for real-time disease surveillance. The aim was to develop a proof-of-principle system that could be deployed for Covid-19 surveillance, or for any disease where there exists spatially-referenced and time-stamped individual-level case data. Most data sources available in "real-time" are positive case (or proxy) outcomes from healthcare system databases, such as hospitalisations, presentations to health services, or contact with public health telephone or internet services (such as NHS Direct in the UK). This contrasts with binomial testing data from large-scale surveys that occur on a less frequent basis (e.g.[14]). The advantage of healthcare system data is that it also usually has patient residential address to enable geolocation of each case. Following previous applications of real-time surveillance systems,[11] we took a geospatial statistical approach to the development of the software and used data on Covid-19 hospitalisations to demonstrate proof-of-principle. The method we describe below was primarily designed and setup to provide reliable predictions of incidence rates, and not for the purposes of estimating the effects of covariates or the nature of spatial and temporal correlation structure. However, outputs from these models provide useful descriptions of disease risk, its variation across an urban area and its correlations with key predictors.

Data

We obtained data on all Covid-19 hospitalisations to hospitals in the University Hospitals Birmingham NHS Foundation Trust (UHB-FT) for the period 1st February 2020 to September 30th 2020, which covers the first wave of Covid-19 in the UK. A Covid-19 hospitalisation was defined as any inpatient admission where the primary diagnosis was Covid-19; we did not specify how the diagnosis was made. Our primary data for these patients comprised their residential address and date of admission.

For peer review only

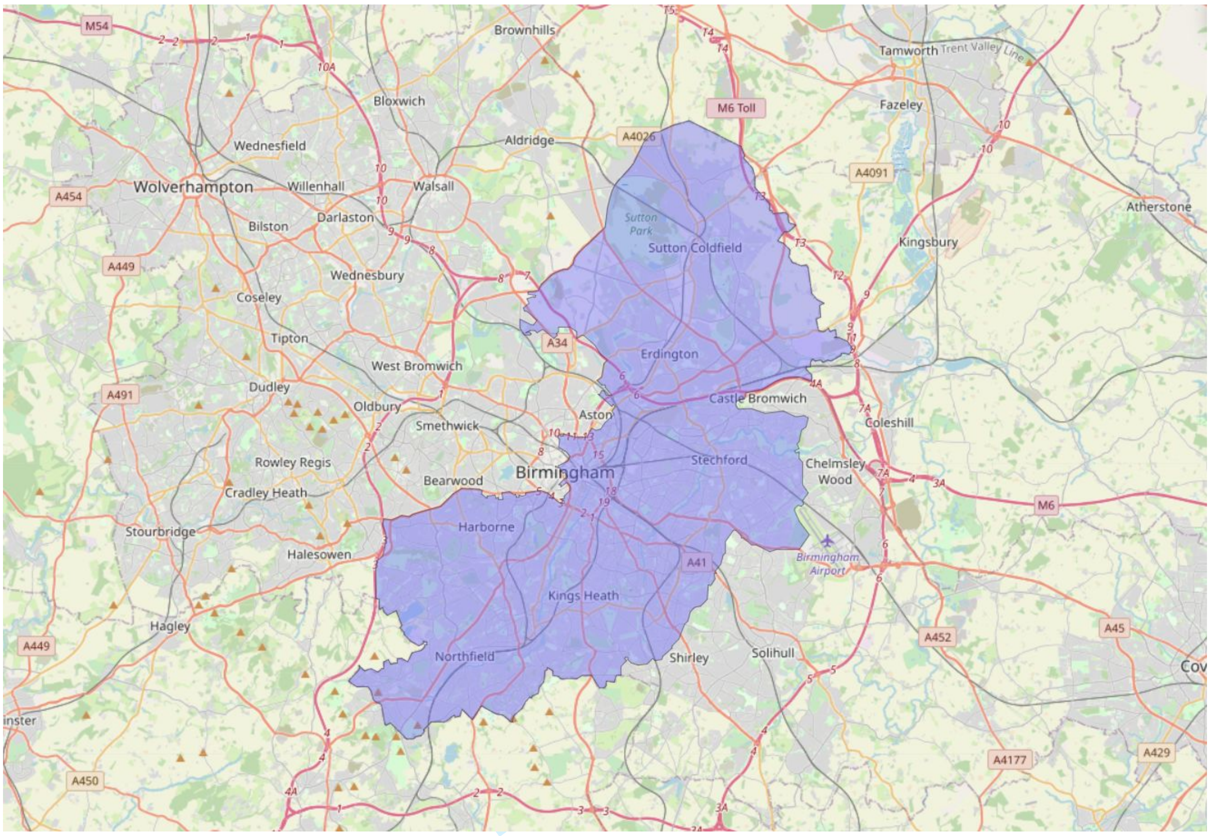

Figure 1. Boundary of the area of study representing the approximate catchment area of UHB-FT in Birmingham, UK.

We defined our area of interest as the approximate catchment area of UHB-FT within Birmingham, which encompasses approximately 70% of the area of the city (Figure 1). Cases resident outside this area were therefore excluded. We conducted the analysis as if the data were provided daily (see below), and each day's analysis included data from the preceding 14 days. Our first day was taken to be March 23rd 2020, when the UK entered its first nationwide lockdown, so any cases admitted prior to March 9th 2020 were excluded from the data. Prior to March 23rd 2020 there were few admissions overall, and most days had zero admissions. Residential addresses were converted into longitude and latitude coordinates using the Google Maps API.

We obtained population and demographic data for Lower-Layer Super Output Areas (LSOA) covering the area of interest from the Office for National Statistics.[15] Each LSOA covers an average population of 700 people; there were 639 LSOAs within our area of interest. For each LSOA we extracted and compiled projected 2019 population density (people per hectare), the proportion of the population aged over 65, the proportion of the population identifying as white ethnicity, and the Index of Multiple Deprivation (IMD), an index used to capture socioeconomic deprivation based on outcomes including education, employment, and crime.[16] The IMD is an ordinal index, and LSOAs are grouped into deciles, which we used in our analysis. We also

determined from the date of admission, the day of the week of the case, which was also used as a covariate in the predictive model.

Statistical model and computational methods

The Appendix gives a more detailed description of our statistical model and associated methods of inference. Here we give an informal summary.

Our statistical model is a spatio-temporal, log-Gaussian Cox process (LGCP, [19]). This model considers the study-region as a spatio-temporal continuum, within which case-incidence at location *s* and time *t* is expressed as

$$\lambda(s,t) = e(s)\exp\left(\beta_0 + \beta_1 x(s,t) \; + Z(s,t)\right).\#(1)$$

In equation (1) the terms within the exponential decompose the spatio-temporal variation of individual-level risk into three multiplicative components, a constant of proportionality $\exp\left(\beta_0\right)$ and two varying terms $\exp\left(\beta_1 x(s,t)\right)$ and $\exp\left(Z(s,t)\right)$. The first of these is a log-linear regression that accounts for variation in risk that can be explained by measured characteristics $x(s,t)$. The second accounts for any residual, unexplained variation, which we represent as the unobserved realisation of a stochastic process. Specifically, we assume that $Z(s,t)$ is a spatially and temporally correlated Gaussian process. The term $e(s)$ is the population density (number per hectare) at *s*, which converts risk into an expected incidence per hectare per unit time; we assume that $e(s)$ does not change materially over the time-window of the data.

To fit the model, we use Bayesian inference to obtain the joint predictive distribution of $\lambda(s,t)$ at all locations *s* and time *t,* given all available data up to and including time *t*. This allows us to calculate and display suitable summaries of the predictive distribution for whatever properties of the complete history of the incidence surface we wish, for example; current incidence; change in incidence from one day (or week) to the next; exceedance of a pre-specified incidence threshold; decomposition of incidence into explained and unexplained components. This flexibility is a crucial aspect of our approach, which allows predictions to be made in whatever form is most relevant to each user's needs.

Although our model is formulated in a spatio-temporal continuum, for computation we approximate the study-region by a grid of square cells with side-length approximately 50 metres, and record the time of each case as an integer number of days since 23rd March 2020.

Presentation of results

For peer review only

The model described in Equation (1) can be used to generate a range of outputs and predictions relevant to disease surveillance for each lattice cell. These include:

(i) Incidence of Covid-19 hospitalisations per 10,000 person-days. We notate this as I_{st} for grid cell s at time t.

(ii) The relative risk of each cell associated with the observed covariates, "Observed RR": $\exp\left(\beta_1 x(s,t)\right)$ in Equation (1).

(iii) The relative risk associated with the Gaussian process "Latent RR": $\exp\left(Z(s,t)\right)$ in Equation (1). This component captures variation in risk associated with unobserved or unexplained local factors.

(iv) The incidence rate ratio (IRR). For each grid cell we determine the incidence per 10,000 person-days relative to the same location 7-days prior: $I_{st}/I_{s,t-7}$

(v) The cumulative incidence up to time T, which is $\sum_{t=1}^{T} I_{st}$.

We graphically examine the posterior means of these outputs for the grid cells. We also report the posterior distributions for the model parameters (both in the linear predictor and covariance function) at different points during the first wave (April 1st, May 1st, and June 1st 2020).

Finally, we also consider the "persistency" of risk across the area of interest. For each day of the analysis we identified the top 10% of lattice cells in terms of population standardized incidence. For each cell we then determined the proportion of days it was in the top 10%. If risk were completely random across the area then each cell should appear in the top 10% approximately 10% of the time. If it were completely deterministic then 10% of cells would appear 100% of the time.

RESULTS

For the period 23rd March to 30th September there were 4,040 recorded admissions for Covid-19 to UHB-FT hospitals. Of these, 2,668 could be geolocated and were resident in the area of interest. Figure 2 summarises the model predictions. Figure 3 provides model geographic outputs for April 1st 2020.

The first wave of the pandemic is evident from days 1 to 50 post-March 23rd, with peak hospitalisations occurring in the first and second weeks of April 2020. A day-of-the-week effect is evident in the incidence, particularly considering the observed relative risk. Table 1 reports estimates of model parameters; the rate of hospitalisations was 15-30% lower at the weekend

For peer review only

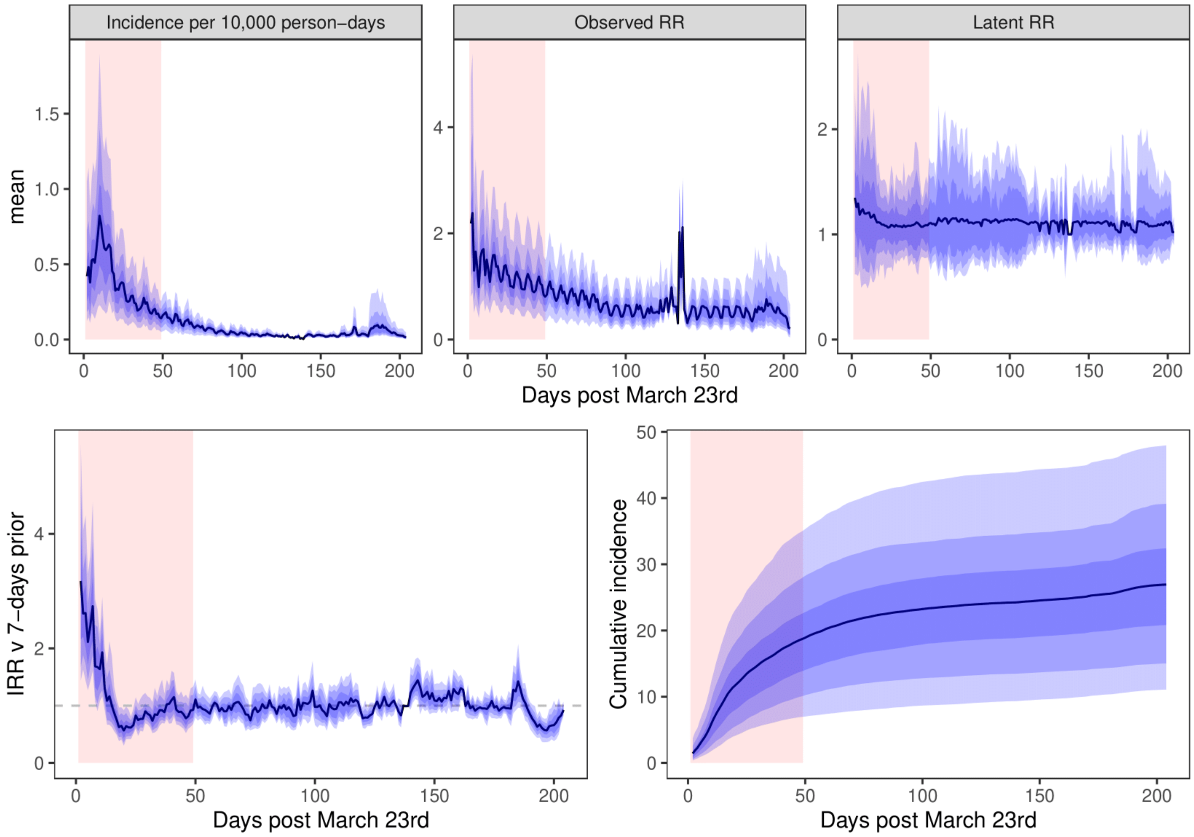

Figure 2. Model predictions of city-wide Covid-19 incidence, relative risks, 7-day incidence rate ratios (IRR), and cumulative incidence. Plots show mean value of posterior mean predictions across the city with bands for 50%, 80% and 95% intervals. The March-May lockdown period is highlighted in red.

than on weekdays. There was significant variation in incidence rates across the city. For example, the 2.5th and 97.5th quantiles of posterior mean incidence rates on March 30th (day 7) differed by a factor of approximately 10, they were 0.14 and 1.11 hospitalisations per 10,000 person-days respectively. This difference is also reflected in the cumulative incidence: by day 50 at the end of the first lockdown period the top 2.5% of cells had experienced five times as many cases per 10,000 population (>35) as the bottom 2.5% (<7).

Figure 2 also shows the 7-day IRR, for which both the mean and median posterior mean remained greater than 1 until day 15 of the lockdown. By day 18 the posterior mean was lower than one for almost all cells. Figure 3 shows that there was significant variation in the IRR across the city – in the north of the city one small area had both relatively high predicted incidence and high IRR. A sudden “spike” in observed relative risk is observed around day 140 when hospitalisation rates approached zero.

For review only

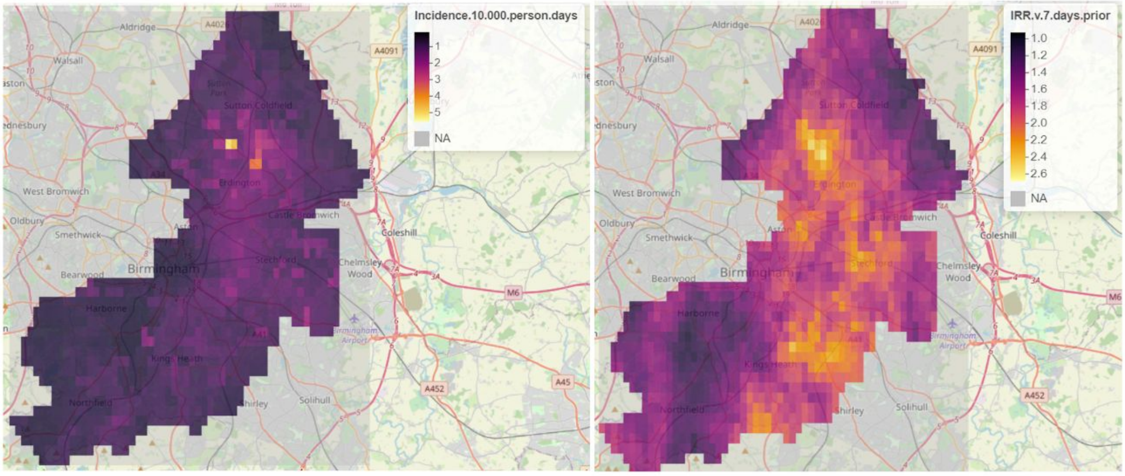

Figure 3. A sample of surveillance system outputs for April 1st 2020. Left: predicted incidence of Covid-19 hospitalisations; right: incidence rate ratio relative to seven-days prior.

Population age, ethnicity, and socioeconomic deprivation were all strongly associated with Covid-19 hospitalisation rates (Table 1). The effect of age was largest in magnitude: a 10-percentage point (pp) increase in the proportion of the population aged over 65 was associated with an approximate doubling in the rate of hospitalisation. A 10 pp increase in the proportion of the population identifying was associated with an approximate 10% reduction in incidence. Good evidence of a 'weekend effect' was also apparent; patients were less likely (~10-20%) to be admitted at the weekend than at the weekday.

As a heuristic, one can multiply the spatial and temporal range parameters by three to get approximate upper limits of the distance and time over which observations are correlated. The spatial correlation was approximately of the order of 0.2km – unexplained increases in incidence were similar across areas of this magnitude. However, the estimated temporal range was substantially shorter than a day. This implies that the unexplained component of local incidence on any one day, $x(s,t)$, is poorly predictive of the unexplained component on the next day. Given the lack of temporal correlation, one might ask whether it is possible to predict the location of local disease clusters given previous data. Figure 4 reports the "persistence" of risk for the 1,411 grid cells across the city. Four grid cells were in the top 10% of grid cells by daily incidence more than 75% of the time. Approximately 5% of grid cells were in the top 10% of grid cells by incidence more than 50% of the time. Approximately 40% of grid cells were never in the top 10%. These results suggest that one could predict a high risk area with reasonable confidence based on its observed characteristics, $x(s,t)$.

For peer review only

Table 1 Posterior mean (95% credible intervals) of model parameters from analyses conducted on three dates.

Parameter	April 1st	May 1st	June 1st
Linear predictor			
10pp increase in proportion of population aged 65 and over	2.12 (1.96, 2.29)	1.86 (1.81, 1.91)	1.72 (1.66, 1.78)
10pp increase in proportion of population identifying as white ethnicity	0.92 (0.90, 0.94)	0.92 (0.91, 0.92)	0.91 (0.90, 0.92)
Increase (less deprived) in IMD decile (linear)	0.93 (0.91,0.95)	0.91 (0.90, 0.92)	0.89 (0.88, 0.90)
Monday	0.99 (0.86, 1.15)	0.85 (0.82, 0.89)	0.98 (0.91, 1.06)
Tuesday	1.10 (0.94, 1.26)	1.01 (0.96, 1.07)	1.09 (1.02, 1.17)
Wednesday	1.12 (0.95, 1.31)	1.12 (1.08, 1.17)	1.11 (1.05, 1.17)
Thursday	0.73 (0.62, 0.86)	1.05 (0.99, 1.12)	0.92 (0.87, 0.97)
Friday	Ref.	Ref.	Ref.
Saturday	0.84 (0.73, 0.80)	0.84 (0.80, 0.89)	0.85 (0.80, 0.90)
Sunday	0.68 (0.58, 0.80)	0.83 (0.79, 0.88)	0.75 (0.71, 0.79)
Covariance parameters			
Sigma	1.12 (1.05, 1.19)	1.05 (1.04, 1.06)	1.05 (1.04, 1.06)
Spatial range	0.07 (0.07, 0.08)	0.07 (0.07, 0.08)	0.08 (0.07, 0.08)
Temporal range	0.16 (0.13, 0.20)	0.11 (0.10, 0.11)	0.10 (0.09, 0.11)

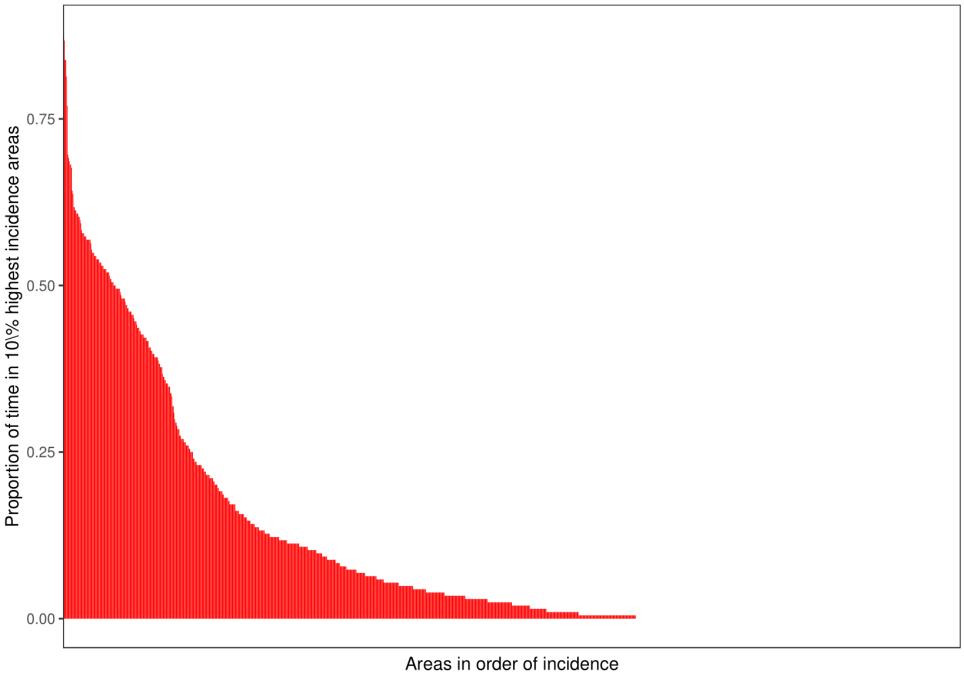

Figure 4. Proportion of time each grid cell was in the highest incidence 10% of grid cells.

DISCUSSION

For peer review

Our results agree with observations made early in the Covid-19 pandemic, namely that age and ethnic minority status is associated with increased risk of Covid-19 transmission.[8,9] Indeed, these factors along with a general measure of socioeconomic deprivation could reasonably predict the small areas of Birmingham with a relatively high incidence of Covid-19 hospitalisations.

We estimated a low temporal correlation in unexplained variation in incidence. This is likely due to the attenuation effect of identifying cases by their date of admission rather than by their (unknown) date of infection, which could have occurred several days or weeks before admission. This is a limitation of many sources of real-time surveillance data, including positive test data. Nevertheless, these results also indicate the strength of our underlying statistical approach, as it enables us to precisely quantify the degree of certainty, or lack thereof, associated with any predictions we make about local disease risk. Crude positive test counts have been the most reported statistic for disease surveillance during the Covid-19 pandemic in the UK and elsewhere.[17] These counts are correlated with the underlying incidence and can provide useful and rapid feedback to officials to implement disease control methods such as lockdowns and tracking and tracing programmes. However, they are a blunt instrument as they do not reflect uncertainty in the underlying estimates of incidence, nor can they offer predictions of locally varying relative risk and associated risk factors. Inferences based only on crude outcomes can easily lead to the identification of signals where none exist.

The results in this article lead to some conclusions to support policy, both specifically for Covid-19 and for disease surveillance in general. First, at the time of writing, vaccination programmes are rolling-out in the UK and elsewhere. A spatial approach should be taken with respect to prioritizing who gets the vaccination. Transmission occurs locally, and areas with older populations, more ethnic minority residents, and that are more socioeconomically deprived have higher rates of transmission. These areas should be prioritized. Second, predicting small-scale day-to-day changes in incidence is difficult and highly uncertain, especially once a pandemic has reached an exponential growth phase. Real-time surveillance systems may have the greatest utility for monitoring diseases that are under general control in the population to enable rapid, targeted responses to emergent disease clusters. Once transmission levels are high across the population there are no longer isolated clusters, although measured characteristics can still give reliable predictions of local relative transmission rates. Third, there are several scientific and statistical approaches to disease surveillance.[18,19] Epidemiological transmission models provide a framework for modelling and estimating transmission rates,[20] but currently available models cannot easily incorporate locally varying and spatially correlated effects. Moreover, these models

For peer review only

often require high-quality data, for example from randomized studies, that can only be collected at relatively infrequent intervals. Geospatial statistical models fitted to electronic health record data offer an alternative method of quantifying uncertainty and making real-time probabilistic predictions that complement other approaches. The methods described in this article use publicly available software and can be run on a desktop machine in a matter of hours using routinely collected data. Thus, there is a strong case for incorporating them into the suite of public health tools used for disease surveillance.

There have been previous examples of proof-of-principle real-time surveillance systems. For example, the AEGISS project used daily data from the then NHS Direct telephone service to identify potential outbreaks of gastro-intestinal disease in Southern England.[4] However, there have been few well-documented implementations of such systems since, despite advances in software and availability of computational resources. Localised spatio-temporal interventions have been trialled for several conditions, such as cholera, and have been broadly successful[20]. Real-time surveillance tools can provide an important and timely input into targeting them.[21]

REFERENCES

1 Bonaccorsi G, Pierri F, Cinelli M, *et al.* Economic and social consequences of human mobility restrictions under COVID-19. *Proc Natl Acad Sci* 2020;**117**:15530–5. doi:10.1073/pnas.2007658117

2 Ratnayake R, Finger F, Azman AS, *et al.* Highly targeted spatiotemporal interventions against cholera epidemics, 2000–19: a scoping review. *Lancet Infect Dis* Published Online First: October 2020. doi:10.1016/S1473-3099(20)30479-5

3 Franch-Pardo I, Napoletano BM, Rosete-Verges F, *et al.* Spatial analysis and GIS in the study of COVID-19. A review. *Sci Total Environ* 2020;**739**:140033. doi:10.1016/j.scitotenv.2020.140033

4 Huang H, Wang Y, Wang Z, *et al.* Epidemic Features and Control of 2019 Novel Coronavirus Pneumonia in Wenzhou, China. *SSRN Electron J* Published Online First: 2020. doi:10.2139/ssrn.3550007

5 Arab-Mazar Z, Sah R, Rabaan AA, *et al.* Mapping the incidence of the COVID-19 hotspot in Iran – Implications for Travellers. *Travel Med Infect Dis* 2020;**34**:101630. doi:10.1016/j.tmaid.2020.101630

6 Ángel Solá DE, Wang L, Vázquez M, *et al.* Weathering the pandemic: How the Caribbean Basin can use viral and environmental patterns to predict, prepare, and

For peer review only

respond to COVID-19. *J Med Virol* 2020;**92**:1460–8. doi:10.1002/jmv.25864

7 Coccia M. Factors determining the diffusion of COVID-19 and suggested strategy to prevent future accelerated viral infectivity similar to COVID. *Sci Total Environ* 2020;**729**:138474. doi:10.1016/j.scitotenv.2020.138474

8 Davies NG, Klepac P, Liu Y, *et al.* Age-dependent effects in the transmission and control of COVID-19 epidemics. *Nat Med* 2020;**26**:1205–11. doi:10.1038/s41591-020-0962-9

9 Sze S, Pan D, Nevill CR, *et al.* Ethnicity and clinical outcomes in COVID-19: A systematic review and meta-analysis. *EClinicalMedicine* 2020;**29–30**:100630. doi:10.1016/j.eclinm.2020.100630

10 Diggle PJ, Giorgi E. Model-Based Geostatistics for Prevalence Mapping in Low-Resource Settings. *J Am Stat Assoc* 2016;**111**:1096–120. doi:10.1080/01621459.2015.1123158

11 Diggle P, Rowlingson B, Su T. Point process methodology for on-line spatio-temporal disease surveillance. *Environmetrics* 2005;**16**:423–34. doi:10.1002/env.712

12 Diggle PJ, Moraga P, Rowlingson B, *et al.* Spatial and Spatio-Temporal Log-Gaussian Cox Processes: Extending the Geostatistical Paradigm. *Stat Sci* 2013;**28**:542–63. doi:10.1214/13-STS441

13 Watson SI. realTimeSurv. GitHub. 2020.

14 Riley S, Ainslie K, Eales O, *et al.* REACT-1 round 6 updated report: high prevalence of SARS-CoV-2 swab positivity with reduced rate of growth in England at the start of November 2020. 2020. https://spiral.imperial.ac.uk/handle/10044/1/83912

15 Office for National Statistics. 2021.https://www.ons.gov.uk/ (accessed 5 Feb 2021).

16 UK Government. English Indices of Deprivation. 2019.https://www.gov.uk/government/statistics/english-indices-of-deprivation-2019

17 UK Government. Coronavirus (Covid-19) in the UK. 2020.https://coronavirus.data.gov.uk/details/cases (accessed 13 Nov 2020).

18 Brookmeyer R, Stroup D. *Monitoring the Health of Populations: Statistical Methods for Public Health Surveillance*. Oxford: : Oxford University Press Inc. 2003.

19 Morton A, Mengersen K, Whitby M, *et al. Statistical Methods for Hospital Monitoring with R*. 2013. doi:10.1002/9781118639153

For peer review only

20 Delamater PL, Street EJ, Leslie TF, *et al.* Complexity of the Basic Reproduction Number (R0). *Emerg Infect Dis* 2019;**25**:1–4. doi:10.3201/eid2501.171901

21 Teng M, Nathoo F, Johnson TD. Bayesian computation for Log-Gaussian Cox processes: a comparative analysis of methods. *J Stat Comput Simul* 2017;**87**:2227–52. doi:10.1080/00949655.2017.1326117

22 Taylor BM, Diggle PJ. INLA or MCMC? A tutorial and comparative evaluation for spatial prediction in log-Gaussian Cox processes. *J Stat Comput Simul* 2014;**84**:2266–84. doi:10.1080/00949655.2013.788653

23 Taylor BM, Davies TM, Rowlingson BS, *et al.* Lgcp: Inference with spatial and spatio-temporal log-gaussian cox processes in R. *J Stat Softw* 2013;**52**:1–40.

24 Taylor BM, Davies TM, Rowlingson BS, *et al.* Bayesian Inference and Data Augmentation Schemes for Spatial, Spatiotemporal and Multivariate Log-Gaussian Cox Processes in R. *J Stat Softw* 2015;**63**. doi:10.18637/jss.v063.i07

For peer review only

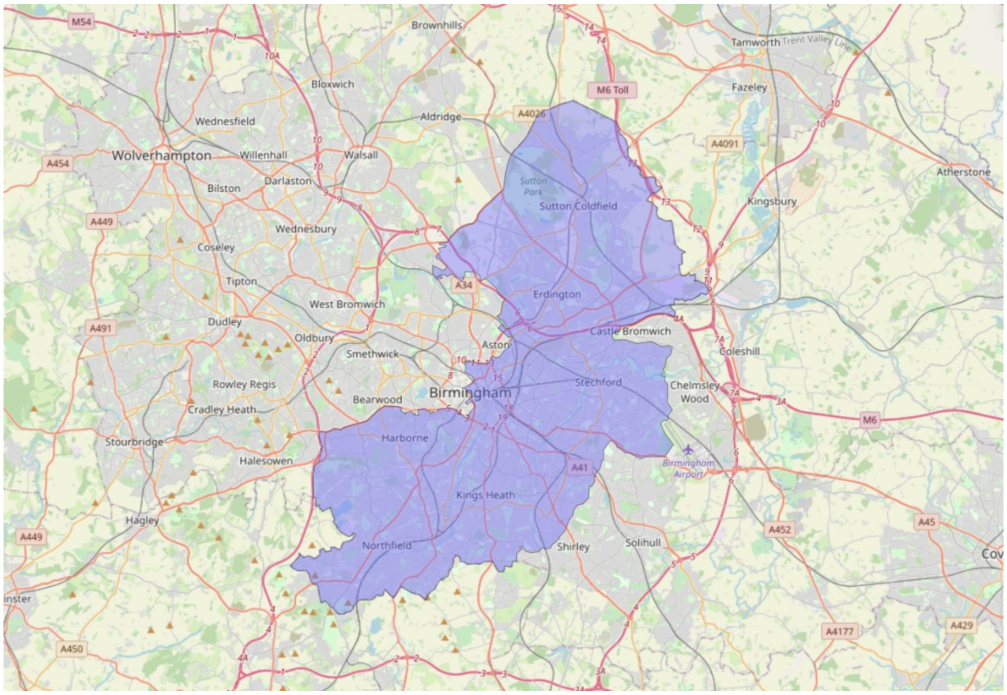

Boundary of the area of study representing the approximate catchment area of UHB-FT in Birmingham, UK.

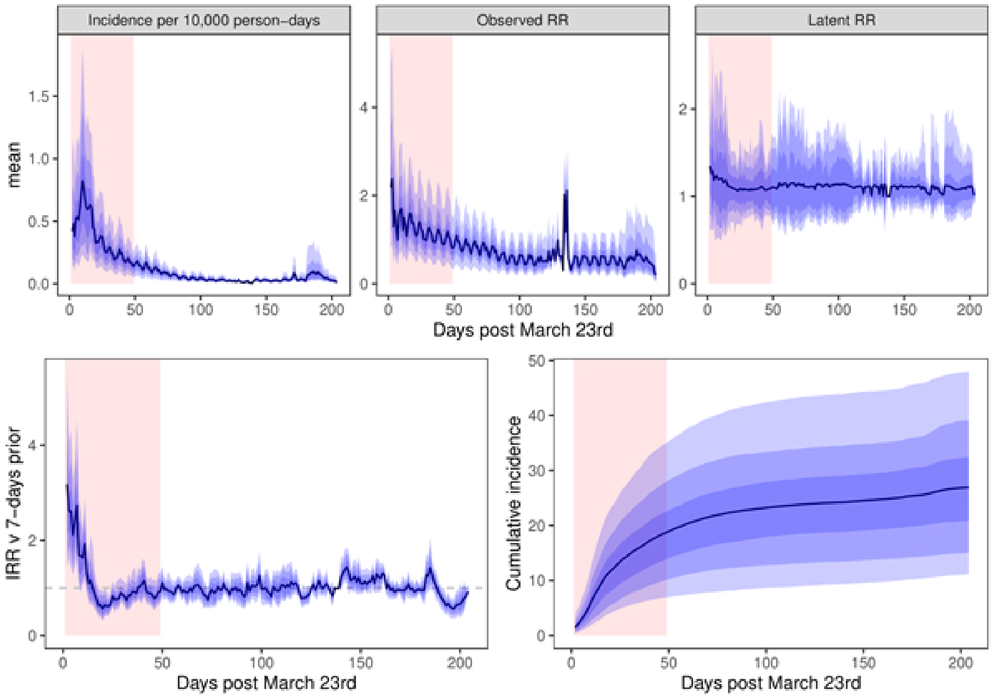

Model predictions of city-wide Covid-19 incidence, relative risks, 7-day incidence rate ratios (IRR), and cumulative incidence. Plots show mean value of posterior mean predictions across the city with bands for 50%, 80% and 95% intervals. The March-May lockdown period is highlighted in red.

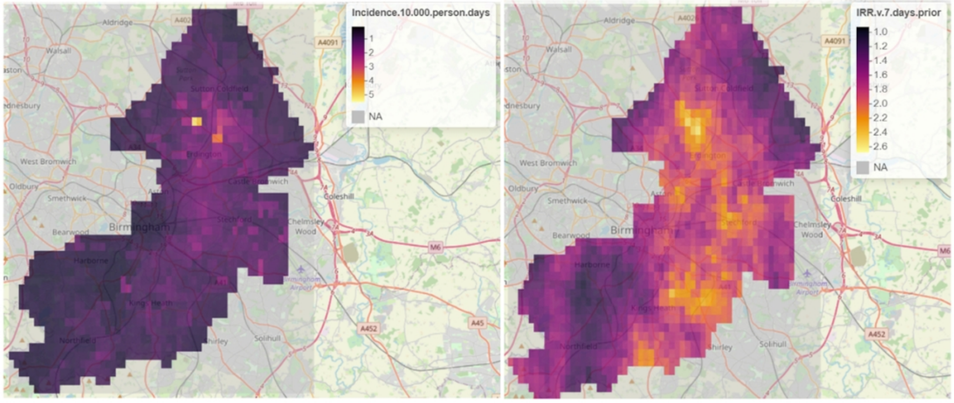

A sample of surveillance system outputs for April 1st 2020. Left: predicted incidence of Covid-19 hospitalisations; right: incidence rate ratio relative to seven-days prior.

SUPPLEMENTARY MATERIAL

The area of interest is $A \subset \mathbb{R}^2$ and the time period of interest is $T \subset \mathbb{R}_{\geq 0}$. The data are assumed to be realisations of a Log Gaussian Cox process (LGCP), i.e. an inhomogeneous Poisson process with stochastic intensity function $\{\lambda(s,t): s \in A, t \in T\}$.[12] The number of cases occurring in any locally finite random set $S \subseteq A$ is Poisson distributed conditional on $\lambda(s,t)$:

$$Y(S,t) = Poisson\left(\int_S \lambda(s,t)ds\right)$$

The intensity of the Poisson process is decomposed as the log-linear model:

$$\lambda(s,t) = e(s)\exp\left(\beta_0 + \beta_1 x(s,t) \ + Z(s,t)\right).\#(1)$$

In equation (1):

1. $e(s)$ is an offset, the population density (number per hectare) at location s; we assumed that changes within the time-window under consideration were negligible.
2. $x(s,t)$ is a set of spatially or temporally varying covariates; no covariates that varied both spatially and temporally on the temporal scale of our analyses were identified.
3. $\{Z(s,t): s \in A, t \in T\}$ is a zero-mean spatio-temporal Gaussian process with minimally parameterised covariance function:

$$Cov\left(Z(s,t), Z(s',t')\right) = \sigma^2 \rho_s\left(||s - s'||;\phi\right)\rho_t\left(||t - t'||;\theta\right)$$

where ||.|| is the Euclidean norm.

We used a double- exponential correlation function:

$$\rho_s\left(||s - s'||;\phi\right) = \exp\left(-\frac{||s - s'||}{\phi}\right);\ \rho_t\left(||t - t'||;\theta\right) = \exp\left(-\frac{||t - t'||}{\theta}\right)$$

where ϕ and θ are the spatial and temporal correlation range parameters.

There are a variety of approaches to fitting LGCPs. Almost all of the computational methods discretize the area of interest using a fine regular lattice onto which case counts are aggregated.[21,22] To ensure the reliability of the discretization, the lattice is generally assumed to be fine enough so that the latent Gaussian field can be assumed to be approximately constant within each cell. A finer lattice would be computationally inefficient, a less fine lattice may smooth over important variation and preclude predictions at finer spatial scales. Based on likely social interactions, disease transmission, and the scales on which variation could be meaningfully interpreted, we selected a cell size of 0.005 on a

For peer review only

longitude/latitude scale, which is approximately $500m^2$. This results in a grid with 1,411 cells inside the boundary of our area of interest.

There have been few head-to-head comparisons of computational methods for LGCPs, and those that do exist consider spatial-models only.[21,22] The principle approaches are Bayesian: a Markov Chain Monte Carlo (MCMC) approach, and a Gauss-Markov Random Field approximation of the model above estimated using MCMC or Integrated Nested Laplacian Approximation. We conducted a scoping comparison of these three approaches using simulated spatio-temporal case data and available software, and in conjunction with previous comparisons, selected a MCMC approach based on the full model as the best performing in terms of stability, computational time, and predictive accuracy. The software package lgcp for R implements the MCMC sampler for this model.[23,24]

For the first day's analysis we used weakly informative $N(0,5^2)$ priors on parameters in the linear predictor and for the log parameters in the covariance function. Weakly informative priors were preferred to "uninformative" priors as they provide a degree of computational stability and regularisation, while providing little information on parameter location within a plausible range for each parameter. For each subsequent day's analysis, the priors were set to the posteriors from the previous day.

For peer review only

BMJ Open

Spatio-temporal analysis of the first wave of Covid-19 hospitalisations in Birmingham, UK

Journal:	*BMJ Open*
Manuscript ID	bmjopen-2021-050574.R1
Article Type:	Original research
Date Submitted by the Author:	26-Aug-2021
Complete List of Authors:	Watson, Samuel; University of Birmingham, Institute of Applied Health Research<br>Diggle, Peter; Lancaster University, Faculty of Health and Medicine; University of Liverpool, Epidemiology and Population Health<br>Chipeta, Michael; University of Oxford<br>Lilford, RJ; University of Birmingham,
**Primary Subject Heading**:	Epidemiology
Secondary Subject Heading:	Health policy, Infectious diseases
Keywords:	COVID-19, Infection control < INFECTIOUS DISEASES, STATISTICS & RESEARCH METHODS

SCHOLARONE™
Manuscripts

I, the Submitting Author has the right to grant and does grant on behalf of all authors of the Work (as defined in the below author licence), an exclusive licence and/or a non-exclusive licence for contributions from authors who are: i) UK Crown employees; ii) where BMJ has agreed a CC-BY licence shall apply, and/or iii) in accordance with the terms applicable for US Federal Government officers or employees acting as part of their official duties; on a worldwide, perpetual, irrevocable, royalty-free basis to BMJ Publishing Group Ltd ("BMJ") its licensees and where the relevant Journal is co-owned by BMJ to the co-owners of the Journal, to publish the Work in this journal and any other BMJ products and to exploit all rights, as set out in our licence.

The Submitting Author accepts and understands that any supply made under these terms is made by BMJ to the Submitting Author unless you are acting as an employee on behalf of your employer or a postgraduate student of an affiliated institution which is paying any applicable article publishing charge ("APC") for Open Access articles. Where the Submitting Author wishes to make the Work available on an Open Access basis (and intends to pay the relevant APC), the terms of reuse of such Open Access shall be governed by a Creative Commons licence – details of these licences and which Creative Commons licence will apply to this Work are set out in our licence referred to above.

Other than as permitted in any relevant BMJ Author's Self Archiving Policies, I confirm this Work has not been accepted for publication elsewhere, is not being considered for publication elsewhere and does not duplicate material already published. I confirm all authors consent to publication of this Work and authorise the granting of this licence.

For peer review only

Spatio-temporal analysis of the first wave of Covid-19 hospitalisations in Birmingham, UK

Samuel I Watson, Senior Lecturer, Institute of Applied Health Research, University of Birmingham

Peter J Diggle, Distinguished Professor of Statistics, Lancaster University

Michael G Chipeta, Research Fellow, University of Oxford

Richard J Lilford, Professor of Public Health, Institute of Applied Health Research, University of Birmingham

Corresponding author

Samuel I Watson

Institute of Applied Health Research, University of Birmingham, Birmingham, B152TT, United Kingdom

s.i.watson@bham.ac.uk

Keywords: Disease surveillance, Covid-19, infectious disease, spatio-temporal modelling

For peer review only

ABSTRACT (243/300 words)

Objectives To evaluate the spatio-temporal distribution of the incidence of Covid-19 hospitalisations in Birmingham, UK during the first wave of the pandemic to support the design of public health disease control policies.

Design A geospatial statistical model was estimated as part of a real-time disease surveillance system to predict local daily incidence of Covid-19.

Participants All hospitalisations for Covid-19 to University Hospitals Birmingham NHS Foundation Trust between February 1st 2020 and September 30th 2020.

Outcome measures Predictions of the incidence and cumulative incidence of Covid-19 hospitalisations in local areas, its weekly change, and identification of predictive covariates.

Results Peak hospitalizations occurred in the first and second weeks of April 2020 with significant variation in incidence and incidence rate ratios across the city. Population age, ethnicity, and socioeconomic deprivation were strong predictors of local incidence. Hospitalisations demonstrated strong day of the week effects with fewer hospitalizations (10-20% less) at the weekend. There was low temporal correlation in unexplained variance. By day 50 at the end of the first lockdown period the top 2.5% of small areas had experienced five times as many cases per 10,000 population as the bottom 2.5%.

Conclusions Local demographic factors were strong predictors of relative levels of incidence and can be used to target local areas for disease control measures. The real-time disease surveillance system provides a useful complement to other surveillance approaches by producing real-time, quantitative and probabilistic summaries of key outcomes at fine spatial resolution to informdisease control programmes.

For peer review only

STRENGTHS AND LIMITATIONS OF THIS STUDY

- Geospatial statistical methods can provide daily predictions of disease epidemiology at small spatial scales as a complement to large-scale designed studies that report infrequently.
- Many countries and local authorities possess the data necessary to run real-time surveillance software that can readily be deployed on a desktop computer, but careful consideration of key model parameters is required.
- Understanding the key predictors and extent of spatial and temporal correlation of disease incidence and transmission can help target local case area targeted intervention (CATI) programmes.
- We report the output of a proof-of-principle surveillance system using geospatial statistical methods to predict local, daily Covid-19 risk using data on hospitalisations for Covid-19 in Birmingham, UK during the first wave of the pandemic in 2020.

For peer review only

ADDITIONAL INFORMATION

Competing interests

None declared.

Funding

UKRI/DHSC COV0036. The funders had no role in study design, data collection and analysis, decision to publish, or preparation of the manuscript.

Author contributions

SW, RJL, PJD, and MGC conceived of the research idea. SW led the development of the software, statistical analyses, and manuscript writing, with support from RJL, PJD, and MGC, who edited and approved the final version of the manuscript.

Ethical approval

Ethical approval for use of the hospital admissions data presented in this article was granted by HRA and HCRW (IRAS 288478).

Transparency declaration

The lead author* affirms that this manuscript is an honest, accurate, and transparent account of the study being reported; that no important aspects of the study have been omitted; and that any discrepancies from the study as planned (and, if relevant, registered) have been explained.

Data sharing statement

Data are not available for sharing from this project. All statistical code and documentation is publicly available at the links provided in the article.

Patient and Public Involvement

Patients were not involved in the design of this study.

Dissemination Declaration

Software and documentation of the detailed methods has been made publicly and freely available.

The Corresponding Author has the right to grant on behalf of all authors and does grant on behalf of all authors, an exclusive licence (or non exclusive for government employees) on a worldwide basis to the BMJ Publishing Group Ltd to permit this article (if accepted) to be published in BMJ editions and any other BMJPGL products and sublicences such use and exploit all subsidiary rights, as set out in our licence.

For peer review only

INTRODUCTION

A range of preventative public health measures have been deployed to limit the transmission of Covid-19 during the pandemic in 2020 and beyond. Perhaps most prominently "lockdowns" of whole regions or nations, involving the closure of businesses and public spaces and requiring people to stay home, have been implemented worldwide. Lockdowns have been effective, but are blunt instruments, with potentially large collateral social and economic effects.[1] More localised approaches that target high-risk areas and emerging disease clusters could potentially ameliorate some of these social and economic effects while maintaining low incidence rates, as has been demonstrated for other infectious diseases. For example, Ratnayake *et al* review the use of case-area targeted intervention (CATI) for cholera, which entails targeting a range of measures at small areas (50-100 meters), including chemoprophylaxis, water treatment, and vaccination, in response to the early detection of an outbreak.[2] However, the effective use of CATI requires both reliable early cluster detection and a good understanding of the spatial and temporal distribution of cases and clusters in order to delineate "case-areas".

There are a growing number of explicit or direct geospatial statistical analyses of Covid-19 transmission and spread.[3] Many of these examples use case data aggregated to small area levels (such as local government authorities or provinces) (e.g.[4,5], see [3] for a review) and so cannot provide insight into the variability of Covid-19 incidence at more local levels, across urban areas. Therefore, there is almost no published evidence or existing reporting systems that can guide CATI-type approaches for Covid-19. There are many "indirect analyses" that compare local or larger-scale risk factors with Covid-19 epidemiology, which could be used to identify high-risk areas for disease control intervention.[3,6,7] For example, population age has consistently emerged as a key risk factor in a number of studies.[8] In high income countries, more socioeconomically deprived areas and areas with a high minority ethnic population proportion have also been identified as high risk.[9] But it is not known just how well these factors explain small-scale spatio-temporal variance in incidence rates.

Geospatial statistical analysis is increasingly in use as a tool for disease surveillance, mapping, and epidemiology.[10] Incident cases and disease testing occur at discrete locations in space and time. Where data on these outcomes are spatially-referenced and time-stamped, they can be modelled to provide predictions of prevalence or incidence over an area of interest. Models can incorporate relevant covariate data and allow for spatial and temporal correlation so that predictions are "smoothed" over time and space. One specific application of these methods is for real-time surveillance.[11] Daily incident case data can be used to predict incidence rates across an area of interest, such as a city, in "real-time" to identify areas

For peer review only

with a high probability of high or rising risk.[12] We developed a proof-of-principle real-time surveillance system using individual-level data on Covid-19 hospitalisations in Birmingham, UK, although the software and statistical approach could be used for any disease. The system is designed to rerun the analyses with each new day's case data, thus its output represents an evolving spatio-temporal analysis of Covid-19 hospitalisations over the course of the pandemic. This article presents results from this analysis for the first wave of the Covid-19 pandemic.

METHODS

The analyses presented in this article were conducted as part of a project to develop software for real-time disease surveillance. The aim was to develop a proof-of-principle system that could be deployed for Covid-19 surveillance, or for any disease where there exists spatially-referenced and time-stamped individual-level case data. Most data sources available in "real-time" are positive case (or proxy) outcomes from healthcare system databases, such as hospitalisations, presentations to health services, or contact with public health telephone or internet services (such as NHS Direct in the UK). This contrasts with binomial testing data from large-scale surveys that occur on a less frequent basis (e.g.[13]). The advantage of healthcare system data is that it also usually has patient residential address to enable geolocation of each case. Following previous applications of real-time surveillance systems,[11] we took a geospatial statistical approach to the development of the software and used data on Covid-19 hospitalisations to demonstrate proof-of-principle. The method we describe below was primarily designed and setup to provide reliable predictions of incidence rates, and not for the purposes of estimating the effects of covariates or the nature of spatial and temporal correlation structure. However, outputs from these models provide useful descriptions of disease risk, its variation across an urban area and its correlations with key predictors. Our software is available online.[14]

Data

We obtained data on all Covid-19 hospitalisations to hospitals in the University Hospitals Birmingham NHS Foundation Trust (UHB-FT) for the period 1st February 2020 to September 30th 2020, which covers the first wave of Covid-19 in the UK. A Covid-19 hospitalisation was defined as any inpatient admission where the primary diagnosis was Covid-19; we did not specify how the diagnosis was made. Our primary data for these patients comprised their residential address and date of admission.

We defined our area of interest as the approximate catchment area of UHB-FT within Birmingham, which encompasses approximately 70% of the area of the city (Figure 1). Cases resident outside this area were therefore excluded. We conducted the analysis as if the data

For peer review only

were provided daily (see below), and each day's analysis included data from the preceding 14 days. Our first day was taken to be March 23rd 2020, when the UK entered its first nationwide lockdown, so any cases admitted prior to March 9th 2020 were excluded from the data. Prior to March 23rd 2020 there were few admissions overall, and most days had zero admissions. Residential addresses were converted into longitude and latitude coordinates using the Google Maps API.

We obtained population and demographic data for Lower-Layer Super Output Areas (LSOA) covering the area of interest from the Office for National Statistics.[15] Each LSOA covers an average population of 700 people; there were 639 LSOAs within our area of interest. For each LSOA we extracted and compiled projected 2019 population density (people per hectare), the proportion of the population aged over 65, the proportion of the population identifying as white ethnicity, and the Index of Multiple Deprivation (IMD), an index used to capture socioeconomic deprivation based on outcomes including education, employment, and crime.[16] The IMD is an ordinal index, and LSOAs are grouped into deciles, which we used in our analysis. We also determined from the date of admission, the day of the week of the case, which was also used as a covariate in the predictive model.

Statistical model and computational methods

The Appendix gives a more detailed description of our statistical model and associated methods of inference. Here we give an informal summary.

Our statistical model is a spatio-temporal, log-Gaussian Cox process (LGCP, [19]). This model considers the study-region as a spatio-temporal continuum, within which case-incidence at location *s* and time *t* is expressed as

$$\lambda(s,t) = e(s)\exp\left(\beta_0 + \beta_1 x(s,t) + Z(s,t)\right).\#(1)$$

In equation (1) the terms within the exponential decompose the spatio-temporal variation of individual-level risk into three multiplicative components, a constant of proportionality $\exp(\beta_0)$ and two varying terms $\exp(\beta_1 x(s,t))$ and $\exp(Z(s,t))$. The first of these is a log-linear regression that accounts for variation in risk that can be explained by measured characteristics $x(s,t)$. The second accounts for any residual, unexplained variation, which we represent as the unobserved realisation of a stochastic process. Specifically, we assume that $Z(s,t)$ is a spatially and temporally correlated Gaussian process. The term $e(s)$ is the population density (number per hectare) at *s*, which converts risk into an expected incidence per hectare per unit time; we assume that $e(s)$ does not change materially over the time-window of the data.

For peer review only - http://bmjopen.bmj.com/site/about/guidelines.xhtml

To fit the model, we use Bayesian inference to obtain the joint predictive distribution of $\lambda(s,t)$ at all locations *s* and time *t,* given all available data up to and including time *t*. This allows us to calculate and display suitable summaries of the predictive distribution for whatever properties of the complete history of the incidence surface we wish, for example; current incidence; change in incidence from one day (or week) to the next; exceedance of a pre-specified incidence threshold; decomposition of incidence into explained and unexplained components. This flexibility is a crucial aspect of our approach, which allows predictions to be made in whatever form is most relevant to each user's needs.

Although our model is formulated in a spatio-temporal continuum, for computation we approximate the study-region by a grid of square cells with side-length approximately 50 metres, and record the time of each case as an integer number of days since 23rd March 2020.

Presentation of results

The model described in Equation (1) can be used to generate a range of outputs and predictions relevant to disease surveillance for each lattice cell. These include:

(i) Incidence of Covid-19 hospitalisations per 10,000 person-days. We notate this as I_{st} for grid cell s at time t.

(ii) The relative risk of each cell associated with the observed covariates, "Observed RR": $\exp\left(\beta_1 x(s,t)\right)$ in Equation (1).

(iii) The relative risk associated with the Gaussian process "Latent RR": $\exp\left(Z(s,t)\right)$ in Equation (1). This component captures variation in risk associated with unobserved or unexplained local factors.

(iv) The incidence rate ratio (IRR). For each grid cell we determine the incidence per 10,000 person-days relative to the same location 7-days prior: $I_{st}/I_{s,t-7}$

(v) The cumulative incidence up to time T, which is $\sum_{t=1}^{T} I_{st}$.

We graphically examine the posterior means of these outputs for the grid cells. We also report the posterior distributions for the model parameters (both in the linear predictor and covariance function) at different points during the first wave (April 1st, May 1st, and June 1st 2020).

Finally, we also consider the "persistency" of risk across the area of interest. For each day of the analysis we identified the top 10% of lattice cells in terms of population standardized incidence. For each cell we then determined the proportion of days it was in the top 10%. If risk were completely random across the area then each cell should appear in the top 10% approximately 10% of the time. If it were completely deterministic then 10% of cells would appear 100% of the time.

For peer review only

RESULTS

For the period 23rd March to 30th September there were 4,040 recorded admissions for Covid-19 to UHB-FT hospitals. Of these, 2,668 could be geolocated and were resident in the area of interest. Figure 2 summarises the model predictions. Figure 3 provides model geographic outputs for April 1st 2020.

The first wave of the pandemic is evident from days 1 to 50 post-March 23rd, with peak hospitalisations occurring in the first and second weeks of April 2020. A day-of-the-week effect is evident in the incidence, particularly considering the observed relative risk. Table 1 reports estimates of model parameters; the rate of hospitalisations was 15-30% lower at the weekend than on weekdays. There was significant variation in incidence rates across the city. For example, the 2.5th and 97.5th quantiles of posterior mean incidence rates on March 30th (day 7) differed by a factor of approximately 10, they were 0.14 and 1.11 hospitalisations per 10,000 person-days respectively. This difference is also reflected in the cumulative incidence: by day 50 at the end of the first lockdown period the top 2.5% of cells had experienced five times as many cases per 10,000 population (>35) as the bottom 2.5% (<7).

Figure 2 also shows the 7-day IRR, for which both the mean and median posterior mean remained greater than 1 until day 15 of the lockdown. By day 18 the posterior mean was lower than one for almost all cells. Figure 3 shows that there was significant variation in the IRR across the city – in the north of the city one small area had both relatively high predicted incidence and high IRR. A sudden “spike” in observed relative risk is observed around day 140 when hospitalisation rates approached zero.

For peer review only

Population age, ethnicity, and socioeconomic deprivation were all strongly associated with Covid-19 hospitalisation rates (Table 1). The effect of age was largest in magnitude: a 10-percentage point (pp) increase in the proportion of the population aged over 65 was associated with an approximate doubling in the rate of hospitalisation. A 10 pp increase in the proportion of the population identifying as white was associated with an approximate 10% reduction in incidence. Good evidence of a 'weekend effect' was also apparent; patients were less likely (~10-20%) to be admitted at the weekend than at the weekday.

As a heuristic, one can multiply the spatial and temporal range parameters by three to get approximate upper limits of the distance and time over which observations are correlated (see Appendix for further statistical details). The spatial correlation was approximately of the order of 0.2km – unexplained increases in incidence were similar across areas of this magnitude. However, the estimated temporal range was substantially shorter than a day. This implies that the unexplained component of local incidence on any one day, $x(s,t)$, is poorly predictive of the unexplained component on the next day. Given the lack of temporal correlation, one might ask whether it is possible to predict the location of local disease clusters given previous data. Figure 4 reports the "persistence" of risk for the 1,411 grid cells across the city. Four grid cells were in the top 10% of grid cells by daily incidence more than 75% of the time. Approximately 5% of grid cells were in the top 10% of grid cells by incidence more than 50% of the time. Approximately 40% of grid cells were never in the top 10%. These results suggest that one could predict a high risk area with reasonable confidence based on its observed characteristics, $x(s,t)$.

For peer review only

Table 1 Posterior mean (95% credible intervals) of model parameters from analyses conducted on three dates.

Parameter	April 1st	May 1st	June 1st
Linear predictor			
10pp increase in proportion of population aged 65 and over	2.12 (1.96, 2.29)	1.86 (1.81, 1.91)	1.72 (1.66, 1.78)
10pp increase in proportion of population identifying as white ethnicity	0.92 (0.90, 0.94)	0.92 (0.91, 0.92)	0.91 (0.90, 0.92)
Increase (less deprived) in IMD decile (linear)	0.93 (0.91,0.95)	0.91 (0.90, 0.92)	0.89 (0.88, 0.90)
Monday	0.99 (0.86, 1.15)	0.85 (0.82, 0.89)	0.98 (0.91, 1.06)
Tuesday	1.10 (0.94, 1.26)	1.01 (0.96, 1.07)	1.09 (1.02, 1.17)
Wednesday	1.12 (0.95, 1.31)	1.12 (1.08, 1.17)	1.11 (1.05, 1.17)
Thursday	0.73 (0.62, 0.86)	1.05 (0.99, 1.12)	0.92 (0.87, 0.97)
Friday	Ref.	Ref.	Ref.
Saturday	0.84 (0.73, 0.80)	0.84 (0.80, 0.89)	0.85 (0.80, 0.90)
Sunday	0.68 (0.58, 0.80)	0.83 (0.79, 0.88)	0.75 (0.71, 0.79)
Covariance parameters			
Sigma	1.12 (1.05, 1.19)	1.05 (1.04, 1.06)	1.05 (1.04, 1.06)
Spatial range	0.07 (0.07, 0.08)	0.07 (0.07, 0.08)	0.08 (0.07, 0.08)
Temporal range	0.16 (0.13, 0.20)	0.11 (0.10, 0.11)	0.10 (0.09, 0.11)

DISCUSSION

Our results agree with observations made early in the Covid-19 pandemic, namely that age and ethnic minority status is associated with increased risk of Covid-19 transmission.[8,9] Indeed, these factors along with a general measure of socioeconomic deprivation could reasonably predict the small areas of Birmingham with a relatively high incidence of Covid-19 hospitalisations. However, further research is required to determine whether these associations persist in subsequent disease waves to guide longer term public health policy.

We estimated a low temporal correlation in unexplained variation in incidence. This is likely due to the attenuation effect of identifying cases by their date of admission rather than by their (unknown) date of infection, which could have occurred several days or weeks before admission. This is a limitation of many sources of real-time surveillance data, including positive test data. Nevertheless, these results also indicate the strength of our underlying statistical approach, as it enables us to precisely quantify the degree of certainty, or lack thereof, associated with any predictions we make about local disease risk. Crude positive test counts have been the most reported statistic for disease surveillance during the Covid-19 pandemic in the UK and elsewhere.[17] These counts are correlated with the underlying

For peer review only

incidence and can provide useful and rapid feedback to officials to implement disease control methods such as lockdowns and tracking and tracing programmes. However, they are a blunt instrument as they do not reflect uncertainty in the underlying estimates of incidence, nor can they offer predictions of locally varying relative risk and associated risk factors. Inferences based only on crude outcomes can easily lead to the identification of signals where none exist.

The results in this article lead to some conclusions to support policy, both specifically for Covid-19 and for disease surveillance in general. First, at the time of writing, vaccination programmes are rolling-out in the UK and elsewhere. A spatial approach should be taken with respect to prioritizing who gets the vaccination. Transmission occurs locally, and areas with older populations, more ethnic minority residents, and that are more socioeconomically deprived have higher rates of transmission. These areas should be prioritized. Second, predicting small-scale day-to-day changes in incidence is difficult and highly uncertain, especially once a pandemic has reached an exponential growth phase. Real-time surveillance systems may have the greatest utility for monitoring diseases that are under general control in the population to enable rapid, targeted responses to emergent disease clusters. Once transmission levels are high across the population there are no longer isolated clusters, although measured characteristics can still give reliable predictions of local relative transmission rates. Third, there are several scientific and statistical approaches to disease surveillance.[18,19] Epidemiological transmission models provide a framework for modelling and estimating transmission rates,[20] but currently available models cannot easily incorporate locally varying and spatially correlated effects. Moreover, these models often require high-quality data, for example from randomized studies, that can only be collected at relatively infrequent intervals. Geospatial statistical models fitted to electronic health record data offer an alternative method of quantifying uncertainty and making real-time probabilistic predictions that complement other approaches. The methods described in this article use publicly available software and can be run on a desktop machine in a matter of hours using routinely collected data. Thus, there is a strong case for incorporating them into the suite of public health tools used for disease surveillance.

We note some weaknesses of our approach. We have cautiously interpreted the parameters associated with local covariates, including age and ethnicity, but all estimates in spatial models are biased to some extent.[21,22] We excluded the data from many admissions, principally because they were out of area, however a significant proportion could not be geolocated. If the missingness of location data were correlated with latent disease risk then this would prejudice our predictions. However, we believe such correlations are likely small. Data quality is one of the key limitations of using routine health system data across all

For peer review only

applications, so results from large, high quality studies should be used to validate any results where possible. Extensions to the type of model used in this article may provide a better fit to the types of data we describe, for example, a Hawkes process model allows for new cases to increase the probability of subsequent cases. However, the greater complexity of such models may require increased computational resources and so limit their usefulness for the types of applications described here. Similarly, alternative approximations may provide a more desirable trade-off between computational time and accuracy.[23] Further research in this area is warranted.

There have been previous examples of proof-of-principle real-time surveillance systems. For example, the AEGISS project used daily data from the then NHS Direct telephone service to identify potential outbreaks of gastro-intestinal disease in Southern England.[11] However, there have been few well-documented implementations of such systems since, despite advances in software and availability of computational resources. Localised spatio-temporal interventions have been trialled for several conditions, such as cholera, and have been broadly successful.[2] Real-time surveillance tools can provide an important and timely input into targeting them.

For peer review only

REFERENCES

1 Bonaccorsi G, Pierri F, Cinelli M, *et al.* Economic and social consequences of human mobility restrictions under COVID-19. *Proc Natl Acad Sci* 2020;**117**:15530–5. doi:10.1073/pnas.2007658117

2 Ratnayake R, Finger F, Azman AS, *et al.* Highly targeted spatiotemporal interventions against cholera epidemics, 2000–19: a scoping review. *Lancet Infect Dis* Published Online First: October 2020. doi:10.1016/S1473-3099(20)30479-5

3 Franch-Pardo I, Napoletano BM, Rosete-Verges F, *et al.* Spatial analysis and GIS in the study of COVID-19. A review. *Sci Total Environ* 2020;**739**:140033. doi:10.1016/j.scitotenv.2020.140033

4 Huang H, Wang Y, Wang Z, *et al.* Epidemic Features and Control of 2019 Novel Coronavirus Pneumonia in Wenzhou, China. *SSRN Electron J* Published Online First: 2020. doi:10.2139/ssrn.3550007

5 Arab-Mazar Z, Sah R, Rabaan AA, *et al.* Mapping the incidence of the COVID-19 hotspot in Iran – Implications for Travellers. *Travel Med Infect Dis* 2020;**34**:101630. doi:10.1016/j.tmaid.2020.101630

6 Ángel Solá DE, Wang L, Vázquez M, *et al.* Weathering the pandemic: How the

Caribbean Basin can use viral and environmental patterns to predict, prepare, and respond to COVID-19. *J Med Virol* 2020;**92**:1460–8. doi:10.1002/jmv.25864

7 Coccia M. Factors determining the diffusion of COVID-19 and suggested strategy to prevent future accelerated viral infectivity similar to COVID. *Sci Total Environ* 2020;**729**:138474. doi:10.1016/j.scitotenv.2020.138474

8 Davies NG, Klepac P, Liu Y, *et al.* Age-dependent effects in the transmission and control of COVID-19 epidemics. *Nat Med* 2020;**26**:1205–11. doi:10.1038/s41591-020-0962-9

9 Sze S, Pan D, Nevill CR, *et al.* Ethnicity and clinical outcomes in COVID-19: A systematic review and meta-analysis. *EClinicalMedicine* 2020;**29–30**:100630. doi:10.1016/j.eclinm.2020.100630

10 Diggle PJ, Giorgi E. Model-Based Geostatistics for Prevalence Mapping in Low-Resource Settings. *J Am Stat Assoc* 2016;**111**:1096–120. doi:10.1080/01621459.2015.1123158

11 Diggle P, Rowlingson B, Su T. Point process methodology for on-line spatio-temporal disease surveillance. *Environmetrics* 2005;**16**:423–34. doi:10.1002/env.712

12 Diggle PJ, Moraga P, Rowlingson B, *et al.* Spatial and Spatio-Temporal Log-Gaussian Cox Processes: Extending the Geostatistical Paradigm. *Stat Sci* 2013;**28**:542–63. doi:10.1214/13-STS441

13 Riley S, Ainslie K, Eales O, *et al.* REACT-1 round 6 updated report: high prevalence of SARS-CoV-2 swab positivity with reduced rate of growth in England at the start of November 2020. 2020. https://spiral.imperial.ac.uk/handle/10044/1/83912

14 Watson SI. rts2 vignette. 2021. http://www.sam-watson.xyz/vignette.html (accessed 24 Aug 2021).

15 Office for National Statistics. 2021.https://www.ons.gov.uk/ (accessed 5 Feb 2021).

16 UK Government. English Indices of Deprivation. 2019.https://www.gov.uk/government/statistics/english-indices-of-deprivation-2019

17 UK Government. Coronavirus (Covid-19) in the UK. 2020.https://coronavirus.data.gov.uk/details/cases (accessed 13 Nov 2020).

18 Brookmeyer R, Stroup D. *Monitoring the Health of Populations: Statistical Methods for Public Health Surveillance*. Oxford: : Oxford University Press Inc. 2003.

For peer review only

19 Morton A, Mengersen K, Whitby M, *et al. Statistical Methods for Hospital Monitoring with R*. 2013. doi:10.1002/9781118639153

20 Delamater PL, Street EJ, Leslie TF, *et al.* Complexity of the Basic Reproduction Number (R0). *Emerg Infect Dis* 2019;**25**:1–4. doi:10.3201/eid2501.171901

21 Hodges JS, Reich BJ. Adding Spatially-Correlated Errors Can Mess Up the Fixed Effect You Love. *Am Stat* 2010;**64**:325–34. doi:10.1198/tast.2010.10052

22 Reich BJ, Hodges JS, Zadnik V. Effects of Residual Smoothing on the Posterior of the Fixed Effects in Disease-Mapping Models. *Biometrics* 2006;**62**:1197–206. doi:10.1111/j.1541-0420.2006.00617.x

23 Solin A, Särkkä S. Hilbert space methods for reduced-rank Gaussian process regression. *Stat Comput* 2020;**30**:419–46. doi:10.1007/s11222-019-09886-w

FIGURE CAPTIONS

Figure 1. Boundary of the area of study representing the approximate catchment area of UHB-FT in Birmingham, UK.

Figure 2. Model predictions of city-wide Covid-19 incidence, relative risks, 7-day incidence rate ratios (IRR), and cumulative incidence. Plots show mean value of posterior mean predictions across the city with bands for 50%, 80% and 95% intervals. The March-May lockdown period is highlighted in red.

Figure 3. A sample of surveillance system outputs for April 1st 2020. Left: predicted incidence of Covid-19 hospitalisations; right: incidence rate ratio relative to seven-days prior.

Figure 4. Proportion of time each grid cell was in the highest incidence 10% of grid cells.

For peer review only

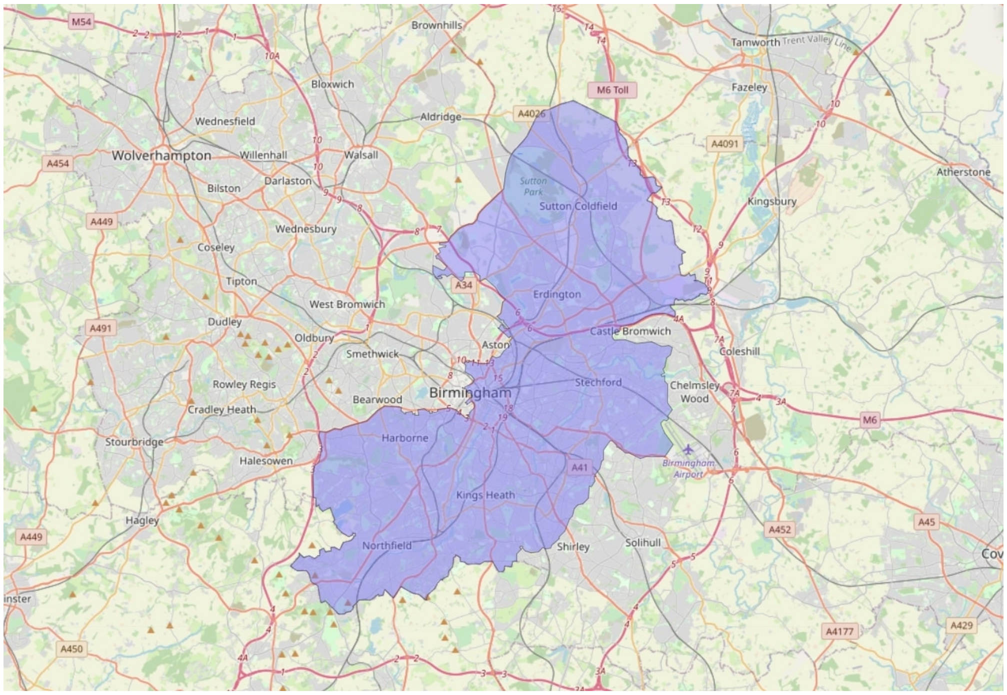

Boundary of the area of study representing the approximate catchment area of UHB-FT in Birmingham, UK.

467x321mm (72 x 72 DPI)

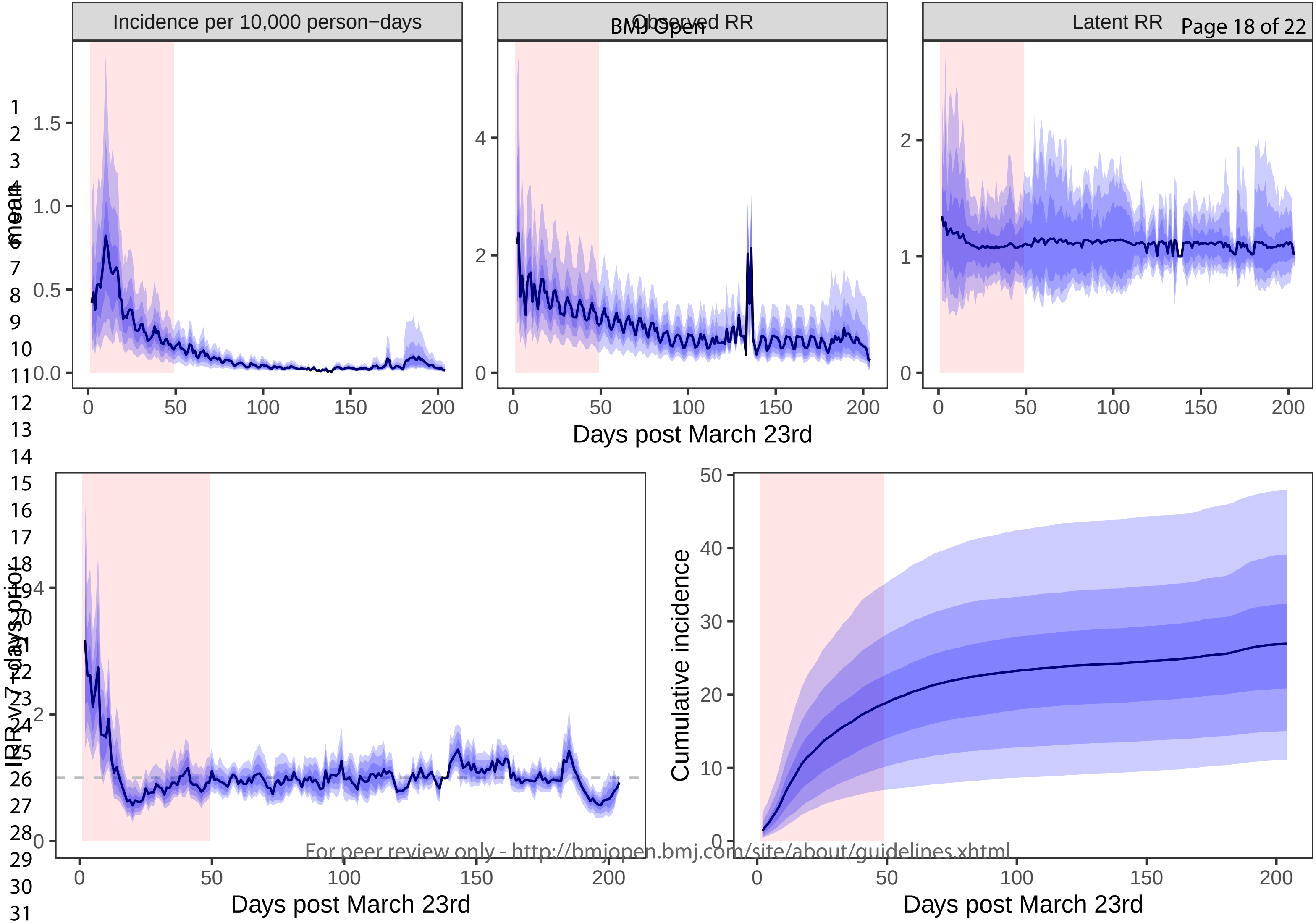
Incidence per 10,000 person−days
Observed RR
Latent RR
mean
Days post March 23rd
IRR vs 7 days prior
Cumulative incidence
Days post March 23rd
Days post March 23rd

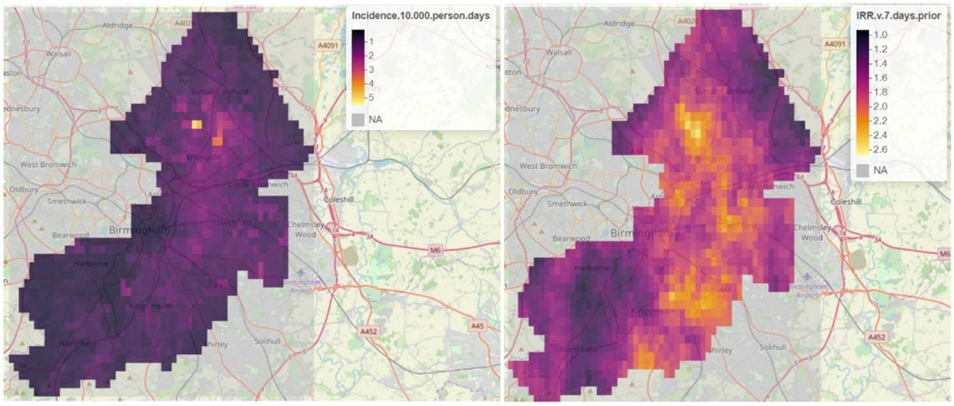

A sample of surveillance system outputs for April 1st 2020. Left: predicted incidence of Covid-19 hospitalisations; right: incidence rate ratio relative to seven-days prior.

477x196mm (72 x 72 DPI)

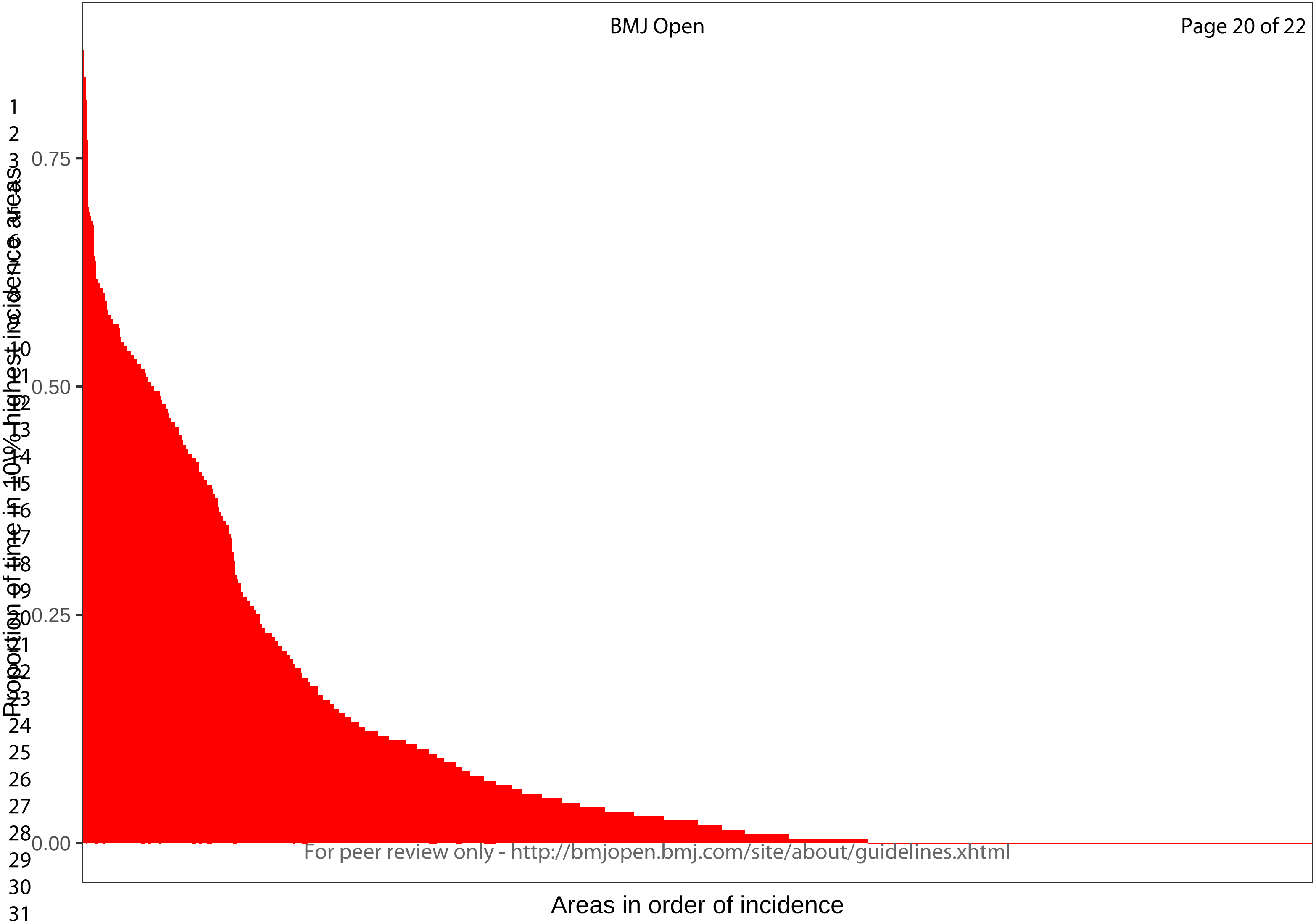
0.75
0.50
0.25
0.00
Proportion of time in 10% highest incidence areas
Areas in order of incidence

SUPPLEMENTARY MATERIAL

The area of interest is $A \subset \mathbb{R}^2$ and the time period of interest is $T \subset \mathbb{R}_{\geq 0}$. The data are assumed to be realisations of a Log Gaussian Cox process (LGCP), i.e. an inhomogeneous Poisson process with stochastic intensity function $\{\lambda(s,t): s \in A, t \in T\}$.[1] The number of cases occurring in any locally finite random set $S \subseteq A$ is Poisson distributed conditional on $\lambda(s,t)$:

$$Y(S,t) = Poisson\left(\int_S \lambda(s,t)ds\right)$$

The intensity of the Poisson process is decomposed as the log-linear model:

$$\lambda(s,t) = e(s)\exp\big(\beta_0 + \beta_1 x(s,t) + Z(s,t)\big). \qquad (1)$$

In equation (1):

1. $e(s)$ is an offset, the population density (number per hectare) at location *s*; we assumed that changes within the time-window under consideration were negligible.
2. $x(s,t)$ is a set of spatially or temporally varying covariates; no covariates that varied both spatially and temporally on the temporal scale of our analyses were identified.
3. $\{Z(s,t): s \in A, t \in T\}$ is a zero-mean spatio-temporal Gaussian process with minimally parameterised covariance function:

$$Cov\big(Z(s,t), Z(s',t')\big) = \sigma^2 \rho_s\big(||s-s'||;\phi\big)\rho_t\big(||t-t'||;\theta\big)$$

where ||.|| is the Euclidean norm.

We used a double- exponential correlation function:

$$\rho_s\big(||s-s'||;\phi\big) = \exp\left(-\frac{||s-s'||}{\phi}\right);\ \rho_t\big(||t-t'||;\theta\big) = \exp\left(-\frac{||t-t'||}{\theta}\right)$$

where ϕ and θ are the spatial and temporal correlation range parameters.

There are a variety of approaches to fitting LGCPs. Almost all of the computational methods discretize the area of interest using a fine regular lattice onto which case counts are aggregated.[1, 2] To ensure the reliability of the discretization, the lattice is

For peer review only

generally assumed to be fine enough so that the latent Gaussian field can be assumed to be approximately constant within each cell. A finer lattice would be computationally inefficient, a less fine lattice may smooth over important variation and preclude predictions at finer spatial scales. Based on likely social interactions, disease transmission, and the scales on which variation could be meaningfully interpreted, we selected a cell size of 0.005 on a longitude/latitude scale, which is approximately $500m^2$. This results in a grid with 1,411 cells inside the boundary of our area of interest.

There have been few head-to-head comparisons of computational methods for LGCPs, and those that do exist consider spatial-models only.[3, 4] The principle approaches are Bayesian: a Markov Chain Monte Carlo (MCMC) approach, and a Gauss-Markov Random Field approximation of the model above estimated using MCMC or Integrated Nested Laplacian Approximation. We conducted a scoping comparison of these three approaches using simulated spatio-temporal case data and available software, and in conjunction with previous comparisons, selected a MCMC approach based on the full model as the best performing in terms of stability, computational time, and predictive accuracy. The software package lgcp for R implements the MCMC sampler for this model.[5, 6]

For the first day's analysis we used weakly informative $N(0,5^2)$ priors on parameters in the linear predictor and for the log parameters in the covariance function. Weakly informative priors were preferred to "uninformative" priors as they provide a degree of computational stability and regularisation, while providing little information on parameter location within a plausible range for each parameter. For each subsequent day's analysis, the priors were set to the posteriors from the previous day.

REFERENCES

1. Diggle PJ, Moraga P, Rowlingson B, Taylor BM. Spatial and Spatio-Temporal Log-Gaussian Cox Processes: Extending the Geostatistical Paradigm. Stat Sci. 2013;28:542–63. doi:10.1214/13-STS441.

2. Møller J, Syversveen AR, Waagepetersen RP. Log Gaussian Cox processes. Scand J Stat. 1998;25:451–82.

3. Teng M, Nathoo F, Johnson TD. Bayesian computation for Log-Gaussian Cox processes: a comparative analysis of methods. J Stat Comput Simul. 2017;87:2227–52. doi:10.1080/00949655.2017.1326117.

For peer review only

4. Taylor BM, Diggle PJ. INLA or MCMC? A tutorial and comparative evaluation for spatial prediction in log-Gaussian Cox processes. J Stat Comput Simul. 2014;84:2266–84. doi:10.1080/00949655.2013.788653.

5. Taylor BM, Davies TM, Rowlingson BS, Diggle PJ. Lgcp: Inference with spatial and spatio-temporal log-gaussian cox processes in R. J Stat Softw. 2013;52:1–40.

6. Taylor BM, Davies TM, Rowlingson BS, Diggle PJ. Bayesian Inference and Data Augmentation Schemes for Spatial, Spatiotemporal and Multivariate Log-Gaussian Cox Processes in R. J Stat Softw. 2015;63. doi:10.18637/jss.v063.i07.

For peer review only
